# Relative influence of inter- and intraspecific competition in an ungulate assemblage modified by introduced species

**DOI:** 10.1093/jmammal/gyad030

**Published:** 2023-03-31

**Authors:** Valentina Zini, Kristin Wäber, Paul M Dolman

**Affiliations:** School of Environmental Sciences, University of East Anglia, Norwich NR4 7TJ, United Kingdom; Natural Capital Solutions, 1 Lucas Bridge Business Park, 1 Old Greens Norton Road, Towcester, Northamptonshire NN12 8AX, United Kingdom; School of Environmental Sciences, University of East Anglia, Norwich NR4 7TJ, United Kingdom; National Trust, Westley Bottom, Westley, Bury Saint Edmunds IP33 3WD, United Kingdom; School of Environmental Sciences, University of East Anglia, Norwich NR4 7TJ, United Kingdom

**Keywords:** feral deer, interference competition, interspecific competition, intraspecific competition, invasion biology, invasive species

## Abstract

Interspecific competition from introduced and naturally colonizing species has potential to affect resident populations, but demographic consequences for vertebrates have rarely been tested. We tested hypotheses of interspecific and intraspecific competition for density, body mass, and fertility of adult female Roe Deer (*Capreolus capreolus*) across a heterogeneous forest landscape occupied by two introduced deer species: Mediterranean Fallow Deer (*Dama dama*); and subtropical Reeve’s Muntjac (*Muntiacus reevesi*). Species-specific deer densities in buffers around culling locations of 492 adult female Roe Deer, sampled over seven years, were extracted from spatially explicit models calibrated through annual nocturnal distance sampling. Roe Deer fertility and body mass were related to species-specific deer densities and extent of arable lands using piecewise structural equation models. Reeve’s Muntjac density was lower at higher Fallow Deer densities, suggesting interspecific avoidance via interference competition, but greater when buffers included more arable land. Roe Deer body mass was marginally greater when buffers included more arable land and was independent of deer densities. However, Roe Deer fertility was unrelated to female body mass, suggesting that fertility benefits exceeded an asymptotic threshold of body condition in this low-density population. However, Roe Deer fertility was slightly greater rather than reduced in areas with greater local Roe Deer density, suggesting negligible intraspecific competition. In contrast, Roe Deer was less fertile in areas with greater Reeve’s Muntjac densities; thus, interspecific exceeded intraspecific competition in this assemblage. In contrast, we found no support for any effects of Fallow Deer density on Roe Deer density, body mass, or fertility. Complex networks of interspecific competition operating in this deer assemblage include: interspecific interference from Fallow Deer exceeded habitat effects for Reeve’s Muntjac; and interspecific competition from introduced, smaller sedentary Reeve’s Muntjac reduced fertility, unlike intraspecific, or potential competition with larger, more mobile, Fallow Deer for native Roe Deer. Mechanisms driving Roe Deer fertility may include interspecific behavioral interference or stress–resource depletion is considered less likely because Roe Deer fertility was independent of body mass. Findings emphasize the importance of ensuring appropriate management strategies for controlling invasive species.

A fundamental principle of species and community ecology is that species cannot stably coexist if the strength of interspecific competition exceeds that of intraspecific competition (Odum 1971; Armstrong and McGehee 1980). Competitively subordinate species are predicted to experience niche reduction, ecological displacement (Hardin 1960; Douglas et al. 1994), or local extirpation if their fundamental niche lies within that of a co-occurring dominant species (Pulliam 2000). Interspecific competition may arise from resource depletion (Dhondt 1977) or interference and agonistic behaviors (Ward and Sutherland 1997; Watts and Holekamp 2008). However, evidence of the effects of interspecific competition remains elusive, as the signature is hard to detect in long-established assemblages, with coexisting species expected to have limited competition through resource partitioning (Hutchinson 1959)–the “ghost of competition past” (Connell 1980). Evidence of interspecific competition is often indirect, inferred through density compensation (e.g., Peres and Dolman 2000) or niche displacement (Herrmann et al. 2021), while demographic consequences are often unresolved (Latham 1999; Dolman and Wäber 2008). The need for greater understanding of the potential strength of interspecific competition is amplified by concern for potential disruption of species assemblages by arriving novel species that evolved in disjunct geographical regions (Mooney and Cleland 2001), with range shifts facilitated by global climatic change and introductions (Walther 2010; Bellard et al. 2012).

Despite frequent concern over the potential of naturally colonizing, introduced or invasive species to impact populations of native species (Mack et al. 2000; Davis 2009; Blackburn et al. 2011; Warren et al. 2016), evidence of interspecific competition is incomplete in contrast to the well-demonstrated impacts of predation (Doherty et al. 2016), disease transmission (Rushton et al. 2006), and ecosystem modification (Hamann 1993; Reaser et al. 2007). Despite strong empirical evidence for interspecific competition in invertebrates (see examples in Schoener 1983; Bengtsson 1989; Human and Gordon 1996), for vertebrates few concrete examples quantify consequences of interspecific competition for demographic parameters (Mishra et al. 2002; Belant et al. 2006; Richard et al. 2009). Competition resulting in habitat, resource (e.g., dietary), or spatial or temporal niche displacement–such as the dietary niche shift of the American Mink (*Mustela vison*) coexisting with recovering native populations of the dominant Eurasian Otter (*Lutra lutra*) (Bonesi et al. 2004), and shifts in temporal activity in foraging bats exposed to other species (Roeleke et al. 2018), can have consequences for individual condition, as in Red Deer (*Cervus elaphus*; Richard et al. 2009). Understanding population consequences of such competition will be strengthened by quantifying demographic responses in relation to the relative densities of native and invading species.

Ungulates are considered keystone species in many ecosystems, as they modify habitat structure and composition, and ecosystem function (McNaughton 1979; Jones et al. 1994; Stewart et al. 2006) with important effects on biodiversity (Crooks 2002; Wright and Jones 2004). Ungulate abundance has increased following landscape modifications, reduced interest in hunting (Ward 2005; Linnell et al. 2020), and in the absence of natural predators across much of North America, Europe (Fuller and Gill 2001; VerCauteren 2003; Côté et al. 2004; Apollonio et al. 2010), and elsewhere (Iijima et al. 2013). Globally, ungulate assemblages are often profoundly modified, with frequent introductions of caprids and cervids across multiple continents (Dolman and Wäber 2008; Genovesi et al. 2012). Intraspecific effects on condition, fertility, and survival have been demonstrated for numerous deer species (Clutton-Brock et al. 1985; Skogland 1985; Richard et al. 2009; Flajšman et al. 2018) but their strength relative to that of interspecific competition is poorly known. To demonstrate competition, demographic parameters of a population need to be measured at varying densities as it has been done in previous studies (Bartmann et al. 1192; McCullough 1979; Kie and White 1985; Houston and Stevens 1988; Hobbs et al. 1996; Stewart et al. 2005). Potential behavioral interference and agonistic interactions may occur between deer species (Forsyth and Hickling 1998; Baldi et al. 2001; Mishra et al. 2002; Stewart et al. 2002; Ferretti 2011; Ferretti, Sforzi, et al. 2011), but demographic consequences are unknown. Co-occurring ungulate species may partition habitat or dietary niche use (Murray and Brown 1993; Singer and Norland 1994; Stewart et al. 2003; Hopcraft et al. 2012), but often show considerable dietary overlap within broad guilds of browsers or grazers (Storms et al. 2008; Obidziński et al. 2013) such that interspecific exploitation competition has been predicted (Dolman and Wäber 2008).

We investigate competition effects for the complex sympatry of native Roe Deer (*Capreolus capreolus*) and two introduced deer species–Fallow Deer (*Dama dama*) and subtropical (SE Asia) Reeve’s Muntjac (*Muntiacus reevesi*). The larger, sexually dimorphic Fallow Deer males weigh between 46 and 93 kg and females weigh between 35 and 56 kg. Although originating in the Mediterranean region, this species is now widely introduced globally (Dolman and Wäber 2008). Reeve’s Muntjac is also sexually dimorphic, with males weighing between 10 and 18 kg and females between 9 and 16 kg; Reeve’s Muntjac have been introduced to the United Kingdom, France, and the Netherlands (Ferretti and Lovari 2014), and have spread rapidly in England where they reach high local densities (Wäber et al. 2013), affecting woodland structure (Ferretti and Lovari 2014). In Roe Deer, intraspecific competition affects reproductive rate (Vincent et al. 1995; Pettorelli et al. 2002), juvenile body mass (Vincent et al. 1995), and neonatal survival (Gaillard et al. 1997). Roe Deer have a lower level of sexual dimorphism and weigh between 10 and 25 kg–being smaller than Fallow Deer, they are considered potentially vulnerable to interspecific competition from Fallow Deer (Hemami et al. 2005; Dolman and Wäber 2008) through agonistic behavior (Ferretti 2011; Ferretti et al. 2011), and spatial avoidance (Ferretti et al. 2011). In addition, Roe Deer could be more negatively influenced by home range size of Fallow Deer than to habitat characteristics alone, and could experience a reduction in body weight at higher Fallow Deer densities (Focardi et al. 2006). Interspecific competition of Reeve’s Muntjac with Roe Deer has been proposed on the basis of dietary and habitat overlap (Dolman and Wäber 2008), but not tested. Fertility consequences for Roe Deer from any or all of these potential interspecific interactions are unknown, and the importance of interspecific relative to intraspecific competition is poorly understood (Elofsson et al. 2017).

We tested a series of a priori hypotheses of intraspecific and interspecific competition of Fallow Deer on Reeve’s Muntjac density; and Fallow Deer and Reeve’s Muntjac on Roe Deer density, body mass, and fertility (Fig. 1). We used a large cull sample of adult female Roe Deer body mass and fertility measures over 7 years (2011–2017) across a heterogeneous and extensive (195 km^2^) forest landscape, across which Fallow Deer density varied along a north–south gradient, and Reeve’s Muntjac density was manipulated quasi-experimentally between forest blocks. Measures were related to annual fine-scale densities of Roe Deer, Reeve’s Muntjac, and Fallow Deer, with resampling of species-specific density surfaces calibrated by intensive annual thermal imaging distance sampling.

**Fig. 1.**
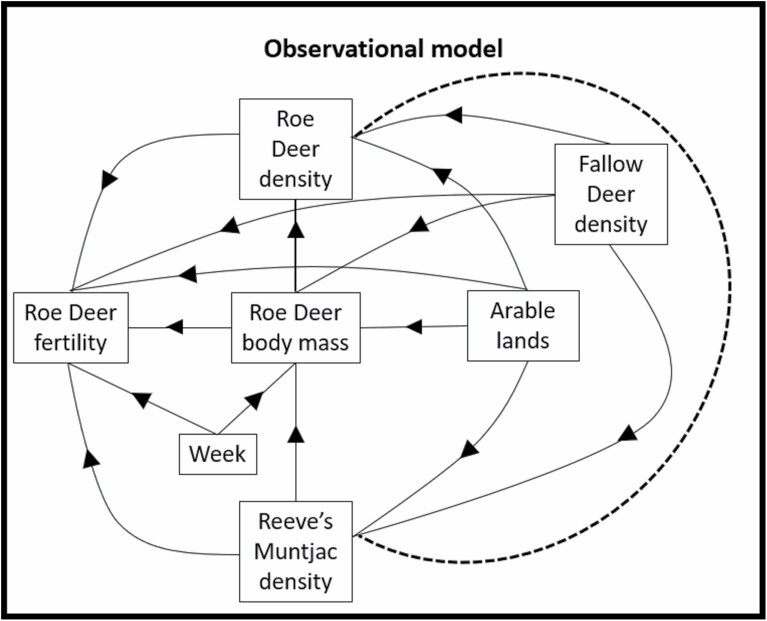
Piecewise structural equation models (SEMs) testing hypotheses of interspecific competition among three deer species, and intraspecific competition of Roe Deer. Observational model representing all hypothesized directional causal effects (arrows) between variables (rectangular boxes, see Table 1 for details) that were tested by SEMs.

**Table 1.** Environmental and deer density variables tested in structural equation models of Roe Deer intraspecific and interspecific competition. For each, the coefficient of variation (*CV*: *SD* as a proportion of the mean) and range are reported, extracted at the mean Akaike-weighted buffer radius around all individual Roe Deer culling locations (pooling across years). DSM = density surface model.

Variable	Description (units)	*CV*	Range	Mean
Arable land	Extent of arable land in the buffer from LCM 2015 (%)	0.9	0–84%	20%
Roe Deer density	Mean Roe deer density across the buffer extracted from year-specific 100-m resolution DSM raster (individual/km^2^)	0.4	1–16	7.4
Reeve’s Muntjac density	Mean Reeve’s Muntjac density across the buffer extracted from year-specific 100-m resolution DSM raster (individual/km^2^)	0.5	5−53	15.1
Fallow Deer density	Mean of Fallow Deer density across the buffer extracted from 3-year composite (sliding mean) 100-m resolution DSM raster (individual/km^2^)	2.3	0–198	1.2

## Materials and Methods

### Study system

Thetford Forest is a conifer-dominated plantation landscape in Norfolk, England, created in the 1930–1950s, and managed by clear-felling harvest and replanting, with much of the forest (62%) now in its second rotation. Across the heterogeneous forest landscape, multiple forest ‘blocks’ (*n* = 14, mean area 1,337 ha, *SD* = 836) provided replication of forest configuration identified as either contiguous core habitat, or outlying blocks with greater access to external arable lands and grassland across Fallow Deer density gradients and soil composition. Within each block, age structure is heterogeneous and fine-grained, comprising even-aged stands (mean area 8.3 ha, *SD* = 5.4) of one of the following types: clear-felled; restocked; pre-thicket; thicket; pole; mature; or overmature stands, including some earlier plantings up to 220 years old. A geographical gradient in Fallow Deer density–combined with variable Roe Deer and Reeve’s Muntjac densities across the forest–provided an opportunity to relate Roe Deer and Reeve’s Muntjac performance to Fallow Deer density, while controlling for interannual variation in weather and local forest composition.

Density dependence and interspecific competition hypotheses (Fig. 1) were examined simultaneously using piecewise structural equation models (SEMs; Shipley 2016), that allow for a quantitative analysis of complex conceptual models, whereby a variable can be both a predictor and a response (unlike in generalized linear models). Roe Deer has a fixed annual fertility cycle (Raganella-Pelliccioni et al. 2007) and cull data were collected in late winter following embryo implantation; yearlings were reliably aged but were excluded from analysis due to low sample size. We a priori examined fertility and body mass responses of adult female Roe Deer to Roe Deer density, species-specific densities of the two introduced deer species, and habitat availability (Fig. 1). In contrast to Roe Deer, we could not reliably model Reeve’s Muntjac fertility as it is an indeterminate aseasonal breeder (Chapman 1991) and cannot be reliably classified into age classes, thus confounding observations. However, we did a priori examine potential responses of local Reeve’s Muntjac density to Fallow Deer density (Fig. 1).

Although SEMs allow multiple hypotheses to be tested simultaneously, reciprocal effects of Reeve’s Muntjac density on Roe Deer density and vice versa could not be considered as the SEM would have been recursive; therefore, we did not examine the potential response of Reeve’s Muntjac to Roe Deer. We also did not examine the response of Fallow Deer to the other species, as Fallow Deer is an invariant breeder. As reviewed in Putman et al. (1996), Fallow Deer reproductive success is not affected by high densities, resource restrictions, climatic variables, or female condition–and fawn rearing is not related to resource quality or population density. Fallow Deer are dominant over both Roe Deer and Reeve’s Muntjac, so we would not expect them to be affected by interspecific competition (Acevedo et al. 2010; Imperio et al. 2012). Potential effects of Red Deer were not examined as these were wide-ranging and pervasive across the entire study landscape, though at a low overall density (mean across the forest, across years = 0.34 individuals/km^2^, *SE* = 0.25).

### Fertility and body mass data

For each of 492 adult female Roe Deer culled, location was recorded using handheld GPS–date, sex, reproductive status (number of corpora lutea, and embryos), and body mass (to the nearest 0.1 kg after head, feet, and viscera were removed, and blood drained by hanging the carcass) were recorded as part of a long-term research collaboration between Forestry England and the University of East Anglia. Høye (2006) showed that using tooth wear and presence or absence of characters on molars and premolars is an accurate to age Roe Deer and we therefore used this method to age individuals. We categorized age of individual as juvenile, yearling, or adult, which was first assessed by experienced rangers before culling the individual, based on a series of body characteristics such as the overall size of the body outline and size of the head and neck compared to the rest of the body (Stubbe 1997). Roe Deer biometrical data were collected in 2011–2017. After the animal was culled, rangers confirmed the first age assessment using a combination of tooth eruption and wear (Aitken 1975; Høye 2006). Carcasses recorded as incomplete (damaged) or considered incomplete (with adult body mass <8 kg; Zini et al. 2019) were excluded from analyses.

Roe Deer embryos are not clearly visible when opening the uterus before early January (Hewison 1996) due to embryonic diapause (Sempere et al. 1998), therefore fertility analyses were restricted to data collected between 1 January and 31 March, and included a fixed categorical effect of week to account for embryo detectability, coded as 0 = weeks 1–3, and 1 = weeks 4–12, following Zini et al. (2019). Fertility in adult Roe Deer was analyzed as the probability of having one or zero versus two embryos, given the scarcity of nonpregnant females (9%) and high frequency of females carrying twin embryos (64%). As the Roe Deer population was heavily culled, senescence with reduced fertility, occurring in females aged 8 years or older (Hewison and Gaillard 2001), was assumed to be negligible, but was confirmed by a deterministic Leslie matrix model that incorporated: study-site-specific measures of yearling fecundity; adult fecundity; neonatal survival from birth to autumn; yearling and adult mortality–estimated from road mortality and culling numbers relative to the estimated population (Supplementary Data SD1)–run to a stable age distribution when females aged ≥8 years comprised only 6% of the prebirthing winter population.

### Deer density mapping

**Fig. 2.**
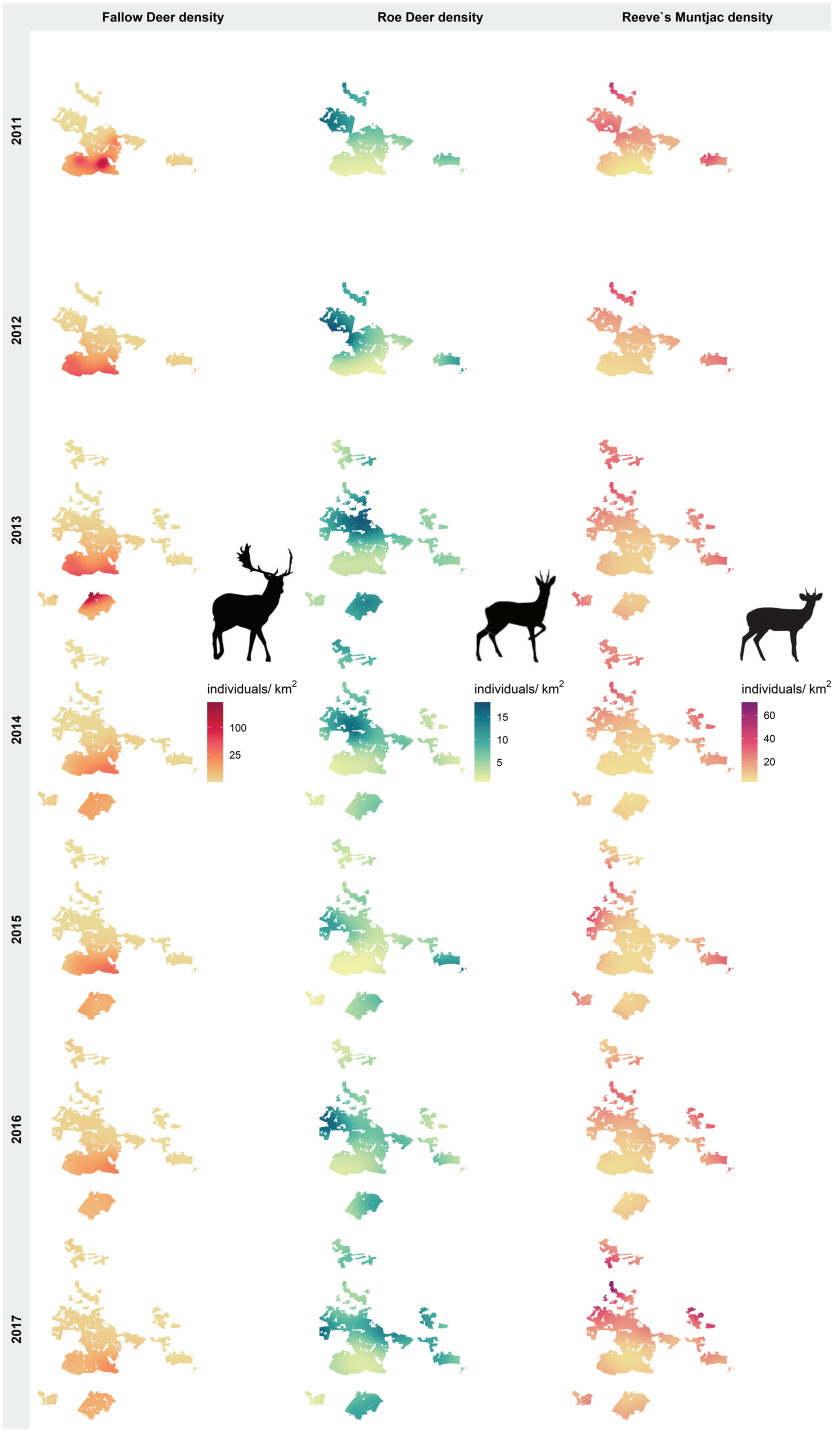
Densities of Reeve’s Muntjac, Roe Deer, and Fallow Deer across Thetford Forest (2011–2017), showing density surfaces (individuals/km2) interpolated at a 100 × 100 m grid from annual density surface models (DSMs) including latitude and longitude. For Fallow Deer, surfaces show the 3-year moving average, due to lower encounter rate, larger group size, and thus greater sampling variance.

In Thetford Forest, densities of Reeve’s Muntjac and Roe Deer vary at a fine scale, with density and activity varying between stands (Hemami et al. 2004, 2005) and population density varying within and between blocks (Wäber et al. 2013; Wäber and Dolman 2015). Culling intensity and pattern varied over the 7 years of the study, including a deliberate effort to experimentally reduce Reeve’s Muntjac numbers in two blocks. However, overall Reeve’s Muntjac numbers increased while Roe Deer numbers fluctuated during the study–both varied in local distribution, further decoupling annual densities from local habitat. In contrast, Fallow Deer range more widely between resting and feeding areas and their density varied at a coarser scale within and between blocks, with a marked density gradient across the landscape, wherein they occurred at high abundance in three southern blocks following landscape-scale colonization, but still absent or scarce in northern blocks (Fig. 2). Throughout the study period Fallow Deer density changed slightly as a management policy was implemented to prevent Fallow Deer numbers from increasing in the southern blocks where Fallow Deer densities were high (Supplementary Data SD2).

For Roe Deer and Reeve’s Muntjac, species-specific deer densities for 2011–2017 were resampled from annual 100-m resolution density surface model (DSM) rasters, generated from distance sampling data obtained by nocturnal thermal imaging transects driven during January to March of each year (Supplementary Data SD3). For Fallow Deer, instead of annual densities, 3-year moving average was calculated due to lower encounter rate, larger group size, and thus greater sampling variance. However, this does not affect our results as Fallow Deer spatial pattern does not change dramatically between years, but we acknowledge this may be a limitation. Distance sampling procedures followed Wäber and Dolman (2015; see also Supplementary Data SD4), while DSM predictions followed Zini et al. (2021). DSMs predicted the spatial variation of animal abundance by a two-step approach (Miller et al. 2013). First, species-specific detection functions were fitted to account for declining detectability with greater perpendicular distance (Buckland et al. 2001) that incorporated a covariate modeling differences in visibility class due to forest stand age (Zini et al. 2021)–when supported during initial model selection, a group size adjustment was applied (Supplementary Data SD5). Subsequently, for each survey-year, the selected species-specific detection function (with visibility covariates) was used to estimate abundance per surveyed transect segment (minimum *n* = 587 in 2011, maximum *n* = 1,239 in 2017; Supplementary Data SD5). A generalized additive model modeled the relation between abundance per segment and complex polynomials of latitude and longitude using a penalized thin-plate regression spline (Supplementary Data SD5).

### Analyses

A piecewise SEM consists of multiple dependent variables and a series of proposed direct and indirect (or mediated) cause–effect relations (Bollen 1989), that are written as a series of regressions (individual models) incorporated into a unique observational SEM (Fig. 1). Reported coefficients partition the variance explained by each structural equation. Recently developed piecewise SEMs (Lefcheck 2016) allow for the inclusion of random effects and non-normal error distributions. Preliminary analysis of each individual model was conducted across buffers to select the best-fitting random effect structure. Through comparison of AICc of competing models using the full fixed-effect structure with different random effect structures, the best-fitting random effect structure was selected for subsequent SEM analysis. Analyses were performed using package ‘piecewiseSEM’ (Lefcheck 2016) in R statistical software (R core Team 2018).

Roe Deer are sedentary, especially over the winter season (Bideau et al. 1993) and were hunted by stalking rather drive-hunting–therefore, it was assumed that the location in which the deer was shot lay within its home range. Individual Roe Deer fertility and body mass were related to environmental and species-specific density variables extracted from the area immediately surrounding cull location. No telemetry data were available to support selecting the most appropriate buffer radius–therefore, following Zini et al. (2019), a series of SEMs were fitted, relating fertility and body mass to arable lands extent and species-specific deer densities extracted at increasing buffer radii from 400 to 600 m at 50-m increments, corresponding to home range sizes ranging from 50 to 113 ha, informed by Roe Deer home ranges in comparable temperate study areas, including: northern Italy, France, and Germany, with a monthly 90% fixed kernel home range of 59 ha (Morellet et al. 2013); and Thetford Forest, with a minimum convex polygon of 114 ha (Chapman et al. 1993). Inference was then based on a model averaging approach (Burnham and Anderson 2002) across this range of potential buffer radii.

For SEM analyses, the same data set needs to be used for each individual model, requiring variable extraction at the same buffer radii around cull locations of both Reeve’s Muntjac and Roe Deer. This was solely based on Roe Deer home range because Roe Deer fertility and body mass were the main focus of this analysis–thus, analyses considering Reeve’s Muntjac density as a predictor–which vary at a smaller grain because Reeve’s Muntjac have smaller home ranges (Chapman et al. 1993)–may deliver a weaker level of confidence in the resulting inference.

Roe Deer fertility was related to species-specific density of the three deer species (extracted from DSMs) and Roe Deer body mass using generalized linear mixed models (GLMMs) with binomial error distribution. Models incorporated: random effects of forest block to control for unmeasured effects of human recreational use, forest management, and residual spatial autocorrelation; cull year to control for winter severity and weather effects on forage availability; fixed effects of calendar period (0 or 1) on embryo detectability (see above); and the local extent of arable lands that provide high-quality forage. Previous analysis of Roe Deer cull data spanning 2002–2015 (Zini et al. 2019) showed that body mass was higher, but fertility was (counterintuitively) lower with greater extent of arable lands. No responses were found to other habitat variables including calcareous soil, grassland, young stands, or mature stands measured as a percentage within the home range of an individual–these variables were therefore omitted from our SEMS. The distribution of arable lands was obtained from the Land Cover Map 2015 (hereafter LCM 2015; Rowland et al. 2017) that maps 23 classes to 0.5-ha resolution, and was extracted from buffers around cull locations (see below) using R statistical software and the packages ‘sp’ (Pebesma and Bivand 2005), ‘rgeos,’ and ‘rgdal’ (Roger et al. 2017).

Roe Deer body mass was related to species-specific deer densities, percentage of arable lands, and calendar week (to control for variation in body mass across time) using GLMMs with normal error distribution, again including a random effect of forest block. Roe Deer density and Reeve’s Muntjac density were each related to Fallow Deer density and percentage of arable lands using GLMMs with normal error, again including random effects of forest block and cull year. As Roe Deer and Reeve’s Muntjac have similar habitat requirements (Hemami et al. 2004), the positive relationship between the two densities (*r* = 0.23, calculated using our SEM data set) driven by habitat selection was controlled by introducing a correlated error structure between Roe Deer and Reeve’s Muntjac density. In order to avoid leverage due to the high number of observations with low values, Fallow Deer density was square rooted when included in the SEMs.

No strong intercorrelation (defined as *r* > 0.78; Freckleton 2002) between the predictor variables arable lands extent and species-specific deer densities was found at any buffer radii in any of the regressions contributing to the SEMs. Variable selection and parameter estimation were performed using multimodel inference (MMI; Burnham and Anderson 2002). All possible combinations of variables (shown in Fig. 1) were built, following MMI procedures (Burnham and Anderson 2002). This process was repeated separately for each buffer radius at which densities were calculated. Resulting SEMs were weighted according to Akaike weights calculated across all models, and across all radii, using package ‘qpcR’ (Spiess 2018). This process was repeated 100 times to incorporate uncertainty in deer density estimates extracted from DSMs, and resampling density from the 95% *CI* of density mapped as the coefficient of variation of the density estimate. Model-averaged coefficients and *CI*s were averaged across these 100 iterations. Variables were considered to be supported if they were included in the 85% model confidence set calculated across all models at all buffers, with a model-averaged parameter *CI* not differing from zero. Consequences of interspecific and intraspecific competition for Roe Deer fertility and body mass were evaluated by predicting fertility from a model including supported variables measured at the Akaike-weighted-mean buffer radius. In order to compare the relative strength of predictor variables, standardized model coefficients scaled by standard deviations are reported. Model performance was evaluated through individual *R*^2^ of each regression with the supported variables extracted at the Akaike-weighted mean buffer radius.

## Results

For adult female Roe Deer, mean body mass was 13.7 kg (*SD* = 1.52) and mean fertility (controlling for week) was 1.44 fetus per adult female (*SD* = 0.05). Across the whole SEM, the Akaike-weighted mean buffer radius was 497 m (*SD* = 81 m).

**Fig. 3.**
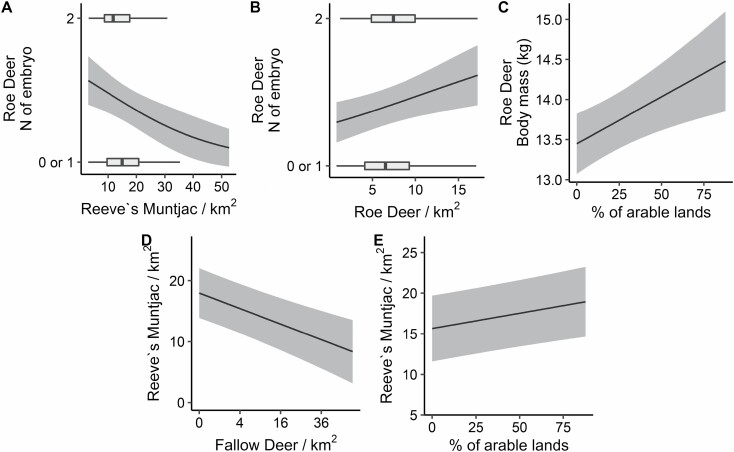
Relation of Roe Deer fertility (A, B), Roe Deer body mass (C), and Reeve’s Muntjac density (D, E), to species-specific local deer densities (A, B, D) and local extent of arable lands (C, E), supported by model averaging across piecewise structural equation models (SEMs). Fertility, body mass, and Reeve’s Muntjac density were predicted from models including variables measured at the Akaike-weighted mean buffer radius (497 m) following model averaging of SEMs across incremental buffer radii (from 400 to 600 m). For fertility, boxplots represent the distribution of individual Roe Deer with either two embryos versus zero or one embryos (combined reference level).

**Fig. 4.**
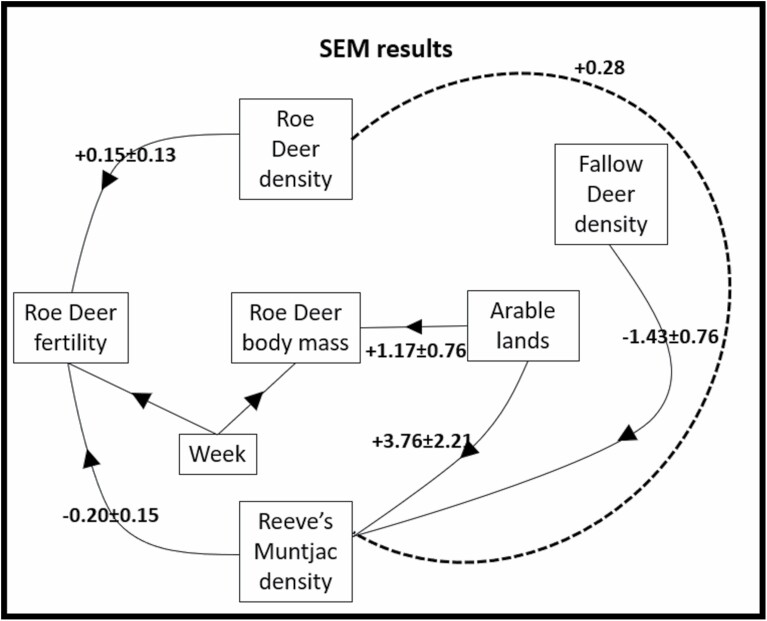
Results of piecewise structural equation models (SEMs) testing hypotheses of interspecific competition among three deer species, and intraspecific competition within Roe Deer, showing supported causal effects (arrows) relating Roe Deer body mass and fertility to intra- and interspecific deer densities and local extent of arable lands, showing model-averaged coefficients (across incremental buffer radii, 400–600 m, corresponding to 59–113 ha) and their 95% confidence interval. Models incorporate random effects of cull year and forest block; dotted line represents the correlated error structure for which the correlation coefficient is also reported.

Reeve’s Muntjac density–at the Akaike-weighted mean buffer radius, individual model *R*^2^ = 0.78–was greater with greater extent of arable lands (interquartile *IQ* = +1.0 individual/km^2^) and lower (*IQ* = 0.79 individual/km^2^) at higher local Fallow Deer density (Figs. 2, 3D, and 3E). The negative effect of Fallow Deer on Reeve’s Muntjac density was stronger than the positive effect of arable lands (Welch’s *t*-test: *t* = 5.5285, d.f. = 597.58, *P* = 4.83e-08). Roe Deer density was unrelated to Fallow Deer density or arable lands extent (with no effects supported; Fig. 4).

The individual model of Roe Deer body mass explained a small proportion of variance (individual *R*^2^ = 0.17), with adult female Roe Deer being heavier in localities with a greater extent of arable lands (*IQ* = +0.32 kg; Figs. 3C and 4). Roe Deer body mass was not related to local Roe Deer density, or to the densities of either Reeve’s Muntjac or Fallow Deer (Fig. 4).

The individual model of Roe Deer fertility explained a small proportion of variance (individual *R*^2^ = 0.10) with lower fertility for individuals culled in buffers with a greater local density of Reeve’s Muntjac (*IQ* = −14% probability of having two embryos instead of one or zero), while fertility was slightly greater in buffers with greater Roe Deer density (*IQ* = +9% probability of having two embryos instead of one or zero; Figs. 3A, 3B, and 4). No effects on Roe Deer fertility of the body mass of an individual, arable lands, or Fallow Deer density were supported (Fig. 4).

## Discussion

This study utilized a large data set, robust measures of deer densities, and a quasi-experimental approach with landscape-scale replication that controlled for forest configuration and habitat quality to investigate interspecific competition between Roe Deer, Reeve’s Muntjac, and Fallow Deer. Although intraspecific competition in Roe Deer has been widely documented elsewhere (e.g., Gaillard et al. 1997; Pettorelli et al. 2002; Douhard et al. 2013), no effects on Roe Deer body mass, or of body mass on individual fertility were found. This is likely due to low overall densities compared to those observed in Thetford in the past or in other forests. However, complex patterns of interspecific competition were found, with apparent displacement of Reeve’s Muntjac but not Roe Deer by Fallow Deer, while Roe Deer were less fertile in areas with higher Reeve’s Muntjac densities, suggesting that interspecific competition with Reeve’s Muntjac outweighed intraspecific competition within Roe Deer in this system.

Competition between introduced Fallow Deer and Reeve’s Muntjac has, to our knowledge, not been investigated previously. We found lower Reeve’s Muntjac densities in localities with more Fallow Deer, with an effect size greater than the positive effect of arable lands. It is plausible that Reeve’s Muntjac may be displaced by the larger, competitively dominant Fallow Deer. Potential displacement may not be neutral in terms of ecosystem structure and function, as cervids can differ markedly in their patterns of seed dispersal (Eycott et al. 2007) and browsing impacts. In contrast, we found no evidence that high densities of Fallow Deer affected Roe Deer density, body mass, or fertility. This lack of interspecific competition is notable, given that Roe Deer are subject to aggression by Fallow Deer (Ferretti 2011) that can displace Roe Deer from feeding areas (Ferretti et al. 2008; Ferretti, Sforzi et al. 2011). In central Italy, Roe Deer densities are negatively related to those of Fallow Deer (Ferretti et al. 2011)–similarly in the United Kingdom, Roe Deer have been reported to be either absent or at low numbers where Fallow Deer occur in high numbers (Carne 1955). For enclosed populations (33.3 km^2^) in central Italy, Fallow Deer decreased Roe Deer habitat quality (Focardi et al. 2006)–and the Roe Deer population decreased while the Fallow Deer population was increasing, suggesting potential negative effects of Fallow Deer on the Roe Deer population (Imperio et al. 2012). It is possible that low overall Roe Deer density, combined with access to external farmland habitats, attenuated Fallow Deer impacts on Roe Deer in our study landscape.

In contrast to the lack of response to Fallow Deer, Roe Deer fertility was lower with higher local Reeve’s Muntjac densities. While potential mechanisms underlying this effect include behavioral interference or exploitation competition through resource depletion, the lack of detectable intraspecific competition in the Roe Deer population (see below) suggests that forage depletion is unlikely. However, physiological and or endocrine stress (indicative of Roe Deer fitness; Escribano-Avila et al. 2013) from agonistic interactions with Reeve’s Muntjac through interference competition may have reduced implantation. Environmental challenges can increase glucocorticoid levels with consequences for decreased fitness (Sapolsky et al. 2000; Gobush et al. 2008; Bonier et al. 2009), and in Roe Deer glucocorticoids have been shown to vary in relation to habitat quality (Escribano-Avila et al. 2013). Interspecific agonistic encounters may also cause physiological stress, for example, the Australian Lace Monitor (*Varanus varanus*) had higher corticosteroid levels when exposed to higher Red Fox (*Vulpes vulpes*) densities (Jessop et al. 2015). Such a proximal effect of Reeve’s Muntjac acting on Roe Deer fertility can explain an earlier counterintuitive finding of apparent lower fertility of Thetford Forest Roe Deer in areas with greater arable lands (Zini et al. 2019). In the current study, inclusion of both Reeve’s Muntjac density and arable lands extent simultaneously within the SEM showed a response to local Reeve’s Muntjac abundance but not to arable lands indicating they may have acted as a proxy for Reeve’s Muntjac density in the earlier study (Zini et al. 2019) that did not incorporate species-specific deer densities. That Roe Deer appeared to be susceptible to competition with Reeve’s Muntjac, but not Fallow Deer, is curious. One possibility is that the mobile nature of Fallow Deer herds and their habit of commuting between diurnal resting and nocturnal feeding areas allowed Roe Deer to avoid them in space and time–in contrast, the highly sedentary nature of local Reeve’s Muntjac home ranges may make them a constant and unavoidable presence within a Roe Deer home range.

The apparent lack of detectable effects of intraspecific competition on Roe Deer body mass or fertility is not unexpected, given low recent overall densities. Annual thermal imaging distance surveys show that Roe Deer abundance in Thetford Forest was reduced, ranging from an average density of 6 to 34 individual/km^2^ per forest block in 2009 (Wäber 2010), down to 4 individual/km^2^ in 2017 (Supplementary Data SD5)–this value is lower than reported elsewhere in southern England, for example, 34–71 individuals/km^2^ (Gill et al. 1996). Previous long-term data from this Roe Deer population showed decrease and subsequent partial recovery of body mass and fertility, attributable to initial population growth from the 1960s to 1990, and subsequent suppression by culling from 2001 to 2009 (Wäber 2010), consistent with intraspecific competition. Elsewhere in Europe, adult female Roe Deer body mass increased by 20% after density decreased by 60% (Pettorelli et al. 2002)–and Roe Deer litter size is density-dependent (Flajšman et al. 2018) but with effects more likely to be detected at high population densities (Gaillard et al. 2000). The maximum number of embryos in Roe Deer is determined by body weight below an asymptotic threshold, after which further increase in body mass does not affect fertility (Flajšman et al. 2017). In the current study, the average adult female body mass of 13.7 kg (*SD* = 1.52) is relatively high compared to a mass of 11.8 kg (*SD* = 2.4) in 1989–1990 when Roe Deer were more abundant (Wäber 2010). An absence of detectable effects of Roe Deer density on body mass is consistent with low overall Roe Deer numbers. Counterintuitively, Roe Deer fertility appeared to be positively–though weakly–related to local Roe Deer density, again implying an absence of intraspecific competition. It is plausible that unmeasured habitat features including low levels of human recreational pressure and composition of ground flora favored both local aggregation and greater fertility.

The results reported here show that Roe Deer potentially experienced interspecific competition from Reeve’s Muntjac, and Reeve’s Muntjac appeared to be displaced by Fallow Deer, but that intraspecific competition was not detected in Roe Deer at the densities investigated. The competitive exclusion principle (Armstrong and McGehee 1980) implies that Roe Deer, Reeve’s Muntjac, and Fallow Deer may not stably coexist, with Roe Deer potentially being excluded to spatially separated refugia. With numbers and range increasing for both Fallow Deer and Reeve’s Muntjac (Noble et al. 2012), competition might be of growing concern for native Roe Deer–however, the effects of competition found in this study were subtle, possibly because of the low population density of Roe Deer. If control measures reduced the introduced species sufficiently for the Roe Deer population to increase to higher abundance, then intraspecific density dependence may assume greater importance relative to interspecific effects. This study provides an example of the complex nature of competitive interactions within a vertebrate guild enhanced by introduced species. We acknowledge that our explanations for the results we found are suggestions of a speculative nature and that further research is needed in this field.

## Supplementary Data

Supplemental data are available at the *Journal of Mammalogy* online.

**Supplementary Data SD1.**—Senescence of adult Roe Deer.

**Supplementary Data SD2.**—Forest-wide density of Fallow Deer.

**Supplementary Data SD3.**—Annual extent of deer sampling by distance sampling transects.

**Supplementary Data SD4.**—Distance sampling thermal imaging surveys.

**Supplementary Data SD5.**—Methodological details of density surface models.

gyad030_suppl_Supplementary_Data_S1

gyad030_suppl_Supplementary_Data_S2

gyad030_suppl_Supplementary_Data_S3

gyad030_suppl_Supplementary_Data_S4

gyad030_suppl_Supplementary_Data_S5

## Data Availability

Data available from Mendeley Data (Zini et al. 2021) DOI: 10.17632/ydws9gnm46.1.
