# Supplementary material for: Relative influence of inter- and intraspecific competition in an ungulate assemblage modified by introduced species
Source: J Mammal. 2023 Mar 31;104(4):879–91. doi: 10.1093/jmammal/gyad030 (PMC10847828; doi:10.1093/jmammal/gyad030)
Supplement: gyad030_suppl_Supplementary_Data_S1 [file gyad030_suppl_supplementary_data_s1.docx]

**Supplementary Data S1: Senescence of adult Roe Deer**

If the Roe Deer population contained a substantial proportion of older females then reproductive senescence (Hewison and Gaillard 2001) could cause additional unexplained variation in observed fertility, lessening the statistical power of environmental models. As the age (in years) of individual adult Roe Deer was not known, the potential age structure of the female population was explored using a structured demographic model. Age-class composition of culled pre-birthing (late-winter) females from larder data was: 19% kids, 8% yearlings, and 73% adults (shooting is random with respect to age). As embryos can be missed shortly after implantation (Ratcliffe and Mayle 1992), mean fertility (from data collected from 2006-2009) was calculated considering females culled after week 6 of each calendar year. Mean yearling fertility was 0.86 (*SD*=0.73) embryos per female per year and for adults 1.44 (*SD*=0.50) embryos per female per year. For 2008-2010, the mean annual mortality rate (assumed to be age-independent), calculated as the proportion of the population (estimated through distance sampling thermal imaging, see Wäber and Dolman, (2015) dying from culling plus recorded road-traffic (RTA) mortality, was estimated at 25% of the population per year (Wäber et al. 2013). Culling is the overwhelming primary cause of mortality, with recorded RTAs forming only a small (5%) proportion of recorded deaths. Although total mortality may be slightly under-estimated due to further unreported RTAs, any bias would result in the proportion of the population comprising older females to be over-estimated. Annual adult survival was calculated considering mortality rate due to culling and RTA (25%). Following Wäber, Spencer, and Dolman (2013) neonatal survival from birth to first winter was calculated by relating the autumn kid-adult female ratio (from field observations in October–November 2007–2009: 83% ± 0.04, 95% *CI*), to average adult fertility from larder data (of January–February 2006–2009). Annual kid survival from birth to the next spring was then calculated considering neonatal mortality (17%) and mortality due to overwinter culling, resulting in a 63% survival probability. A deterministic Leslie matrix was created with a starting population distributed according to the observed age class proportions of kid: yearling: adult, initially assuming all adults were 2 years-old. A stable age composition was reached after 14 years at which point the percentage of senescent individuals (defined as 8 years or older, following (Hewison and Gaillard 2001) comprised only 6% of the female pre-birthing adult winter numbers. We therefore consider that senescence will have only a minor effect on observed adult fertility. Furthermore, as culling is pervasive across the forest, age structure was considered spatially non-confounding.

**Literature Cited**

Hewison A.J.M., Gaillard J.M. 2001. Phenotypic quality and senescence affect different components of reproductive output in roe deer. Journal of Animal Ecology. 70:600–608.

Ratcliffe P.R., Mayle B.A. 1992. Roe deer biology and management. Forestry Commission Bulletin. 105:1–28.

Wäber K., Dolman P.M. 2015. Deer abundance estimation at landscape-scales in heterogeneous forests. Basic and Applied Ecology. 16:610–620.

Wäber K., Spencer J., Dolman P.M. 2013. Achieving landscape-scale deer management for biodiversity conservation: the need to consider sources and sinks. Journal of Wildlife Management. 77:726–736.
