# Supplementary material for: Relative influence of inter- and intraspecific competition in an ungulate assemblage modified by introduced species
Source: J Mammal. 2023 Mar 31;104(4):879–91. doi: 10.1093/jmammal/gyad030 (PMC10847828; doi:10.1093/jmammal/gyad030)
Supplement: gyad030_suppl_Supplementary_Data_S2 [file gyad030_suppl_supplementary_data_s2.docx]

**Supplementary Data S2: Forest-wide density of Fallow Deer**

Since 2014-2015 wildlife management has focussed on reducing the Fallow Deer density in the southern forest blocks. Our DSMs are built independently for each year and highlight areas of high Fallow Deer density pre 2014 in the southern forest and subsequent reduction in Fallow Deer in the same areas, supporting the validity of our models.


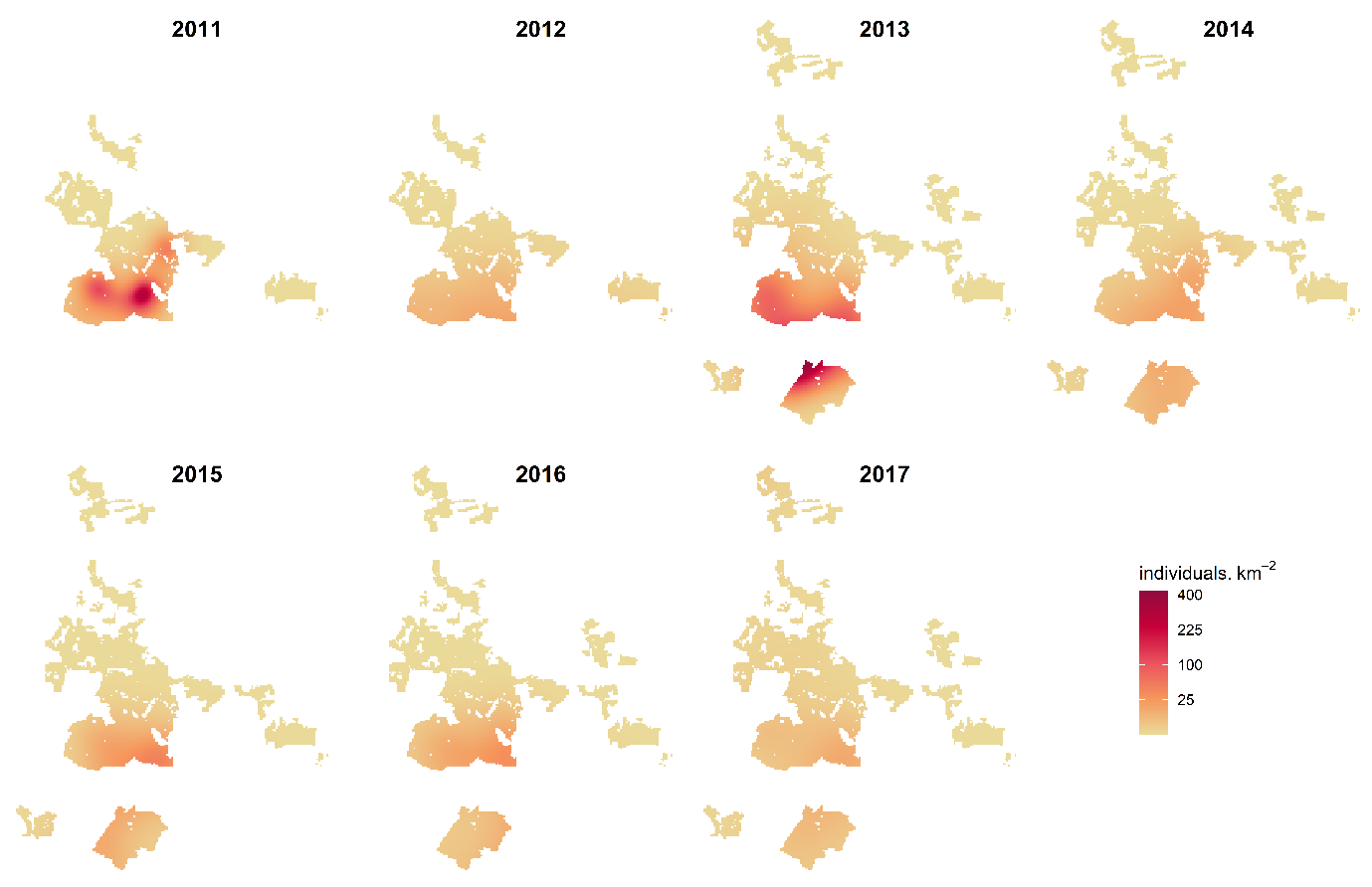


Figure S1: Annual Fallow Deer density across Thetford Forest 2011-2017. Shown are density surfaces (individuals/km^2^) predicted at a 100x100m resolution from density surface models including latitude and longitude.


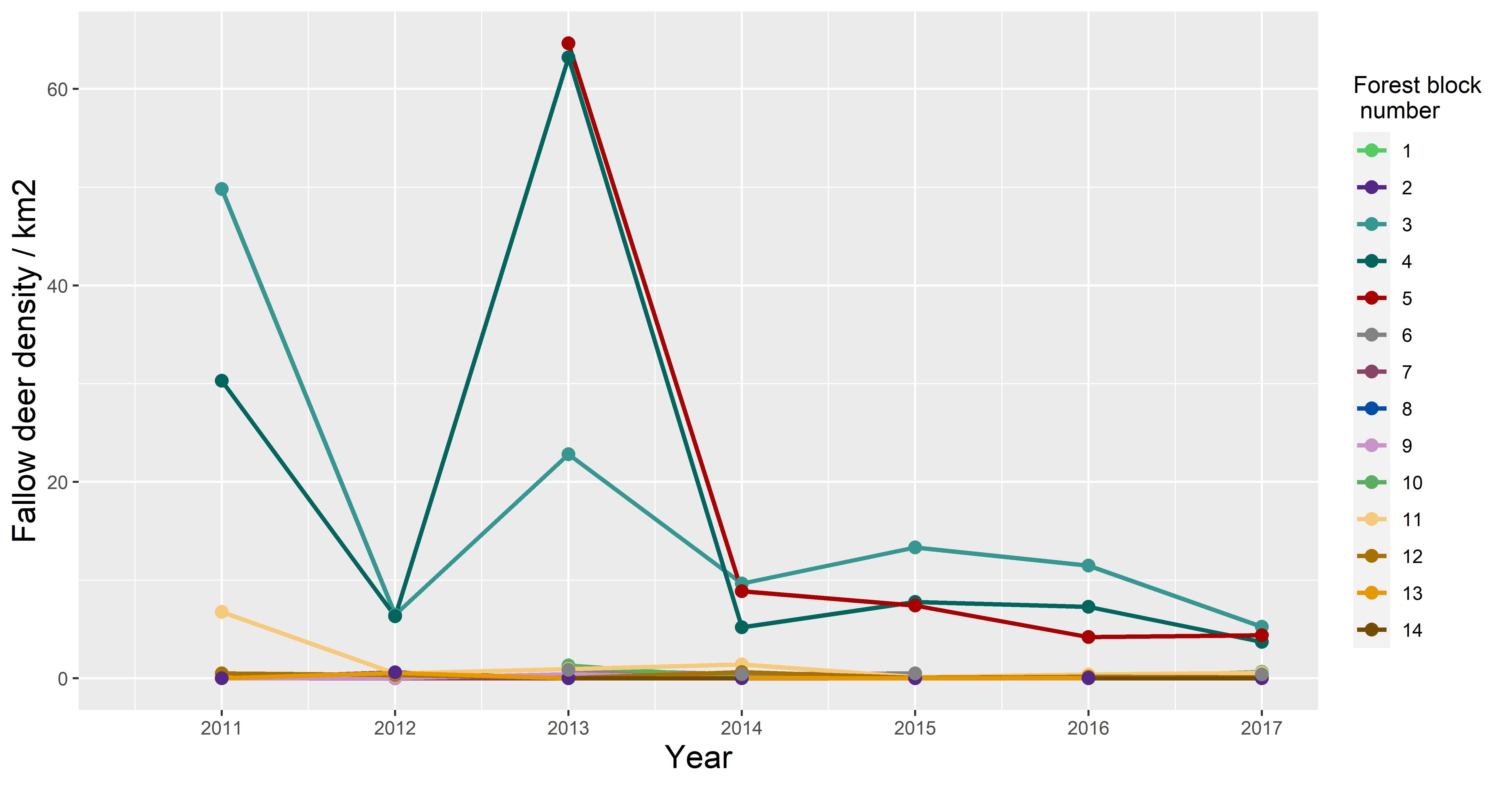


Figure S2: Annual Fallow Deer density per forest block across Thetford Forest 2011-2017. Shown are average Fallow Deer density per forest block (individuals/km^2^).
