# Supplementary material for: Relative influence of inter- and intraspecific competition in an ungulate assemblage modified by introduced species
Source: J Mammal. 2023 Mar 31;104(4):879–91. doi: 10.1093/jmammal/gyad030 (PMC10847828; doi:10.1093/jmammal/gyad030)
Supplement: gyad030_suppl_Supplementary_Data_S3 [file gyad030_suppl_supplementary_data_s3.docx]

**Supplementary Data S3. Annual extent of deer sampling by distance sampling transects**

**
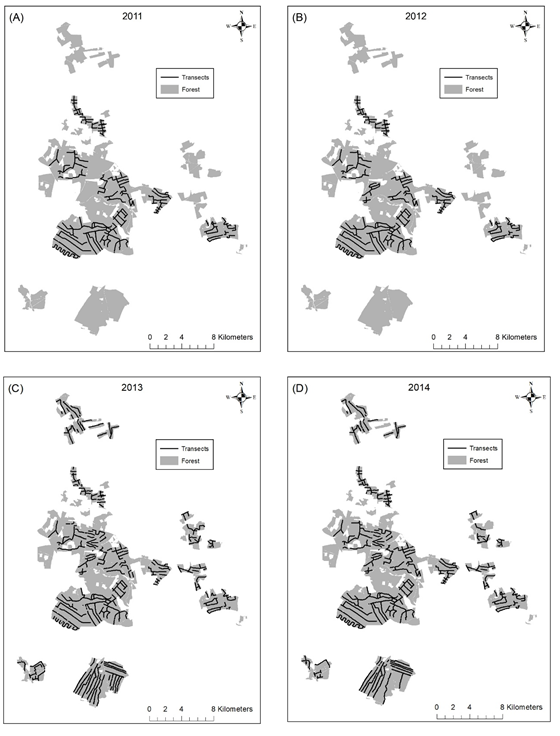
**

**
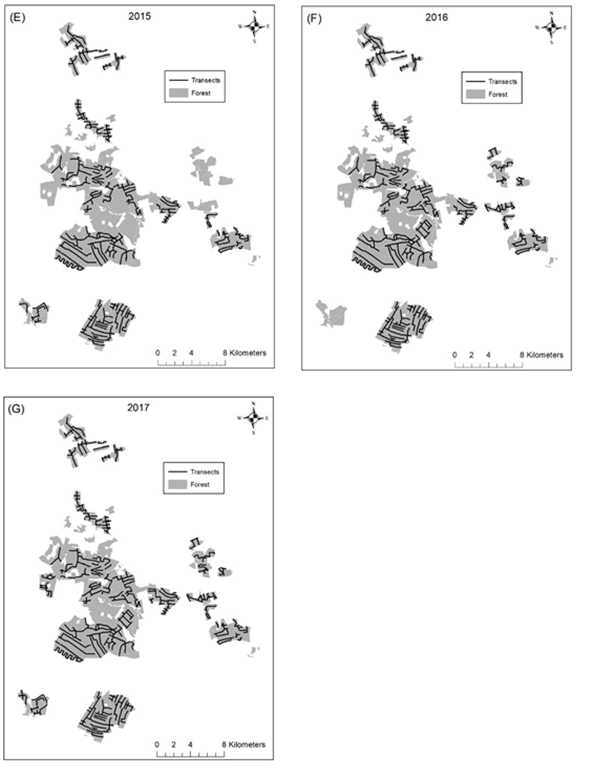
**

Figure S1: Annual thermal imaging distance sampling transects in Thetford Forest from 2011 to 2017. In each year, between 7 and 14 discrete forest blocks were surveyed and density was only estimated for the surveyed forest blocks. For details of transect length and segments, see Table S1.
