# Supplementary material for: Relative influence of inter- and intraspecific competition in an ungulate assemblage modified by introduced species
Source: J Mammal. 2023 Mar 31;104(4):879–91. doi: 10.1093/jmammal/gyad030 (PMC10847828; doi:10.1093/jmammal/gyad030)
Supplement: gyad030_suppl_Supplementary_Data_S4 [file gyad030_suppl_supplementary_data_s4.docx]

**Supplementary Data S4: Distance sampling thermal imaging surveys.**

Distance sampling data were collected at night during the late winter as died-back vegetation allowed better detectability of animals and lower temperatures increase thermal contrast. One-sided transects were conducted from the front passenger seat of a 4-wheel drive a vehicle, driven at a maximum speed of 16 km/h with headlights off. The distance to each individual (or when a group was observed, the distance to the centre of the group) was measured using a Leica Laser Range Finder ‘LRF 800’ that was rigidly mounted on a night vision ‘Maxi-Kite Mk 4’ (THALES optics, St. Asaph, UK) with an infrared illuminator. An angle board was used to estimate (to 5^◦^) the angle of the animal to the transect line to allow subsequent calculation of perpendicular distance.
