# Supplementary material for: Relative influence of inter- and intraspecific competition in an ungulate assemblage modified by introduced species
Source: J Mammal. 2023 Mar 31;104(4):879–91. doi: 10.1093/jmammal/gyad030 (PMC10847828; doi:10.1093/jmammal/gyad030)
Supplement: gyad030_suppl_Supplementary_Data_S5 [file gyad030_suppl_supplementary_data_s5.docx]

**Supplementary Data S5: Methodological details of density surface models**

Density surface models (DSMs) predict the spatial variation of animal abundance by a two-step approach following (Miller et al. 2013); first, species-specific detection functions to account for declining detectability with greater perpendicular distance (Buckland et al. 2001) were fitted in order to select the key detection function, also incorporating a detectability class covariate (three levels: open, dense, and mature) modelling differences in visibility class due to forest stand age, and whether or not the stand had been thinned (see below for details on detectability class covariate). Separately for each survey year, the selected species-specific detection function and covariate were fitted, giving estimated abundance per surveyed transect segment (Table S1). Distance sampling data were recorded from one sided transects with the opposite side of the transect being surveyed the same night and while we acknowledge that individuals might be disturbed by the vehicle during the first visit resulting in an underestimation of density, Zini et al. (2022) shows visitation order doesn’t affect deer density estimation. The lower number of individuals (especially for Roe Deer) close to the transect is not to be interpreted as disturbance cause by the vehicle, rather as slight trackway avoidance which would not significantly bias density estimation (Wäber and Dolman 2015). If our density estimation was biased due to distance sampling being carried out on trackways, this would imply that our density estimates are lower compared to the real density of deer; this bias would affect the whole dataset equally and still not affect our SEM analysis. A Generalised Additive Model (GAM) modelled the relation between abundance per segment and latitude and longitude as a smoothing parameter using a penalised thin plate regression spline.

Table S1: Annual thermal imaging distance sampling transects in Thetford Forest from 2011 to 2017, showing for each year: the numbers of forest blocks surveyed, numbers of distance transect segments used in the density surface model, mean survey effort (transect distance relative to total block area) across surveyed blocks, and total length of transects driven .

| Year | Number of blocks surveyed | Number of segments | Transect driven (km) | Mean effort (km/km^2^) |
| --- | --- | --- | --- | --- |
| 2011 | 7 | 587 | 145.7 | 1.11 |
| 2012 | 7 | 604 | 150.5 | 1.14 |
| 2013 | 13 | 1075 | 264.8 | 1.41 |
| 2014 | 13 | 1078 | 264.0 | 1.41 |
| 2015 | 12 | 1013 | 245.2 | 1.47 |
| 2016 | 12 | 1075 | 264.5 | 1.45 |
| 2017 | 14 | 1239 | 299.8 | 1.60 |

*Detection function, covariate, and adjustments selection*— Analyses were conducted separately for each species, using data pooled across 2011 to 2017. We evaluated alternative detection function models fitting each of the available key detection functions: half-normal and hazard-rate with adjustments (cosine, hermite, polynomial), with and without a term relating group size to perpendicular distance (to account for potentially lower detectability of distant smaller groups), and with or without the categorical detectability class covariate. Following (Buckland et al. 2001) the 5% most distal observations were excluded. The model with the lowest value of Akaike’s information criterion (AIC) was selected for each species, unless alternative models differed by less than 2 units when the most parsimonious model was also accepted. Analyses were performed in R (R core Team 2018) using package “Distance” (Miller 2016).

For Reeve’s Muntjac and Roe Deer, a hazard-rate detection function with detectability class and group size covariates were superior to alternative formulations (Table S2, Fig S2). Despite the smaller number of Fallow Deer observations compared to Roe Deer, or Reeve’s Muntjac (see Table S3), alternative detection functions fitted the data well, but had similar fit (Table S2, Fig S2). We selected: a hazard-rate detection function with detectability class covariate and no support for group size covariate (Table S2, Fig S2). Hazard-rate detection function was selected for Fallow Deer as it limits the potential bias of transect avoidance (see Elenga et al. 2020). A more parsimonious model with similar degree of support (based on AIC) but that lacked the detectability class covariate was rejected as detectability class was considered necessary, as extensive fieldwork experience confirmed age class profoundly affects the ability to detect even large deer.

Table S2: Detection function, covariate, and adjustment selection of Reeve’s Muntjac, Roe Deer, Fallow Deer distance sampling data. For each model AIC, and the difference in AIC value relative to the best supported model (∆AIC) are shown

| Reeve’s Muntjac | | | |
| --- | --- | --- | --- |
| Key function | Formula | AIC | ∆AIC |
| Hazard-rate | ~detectability class + group size | 21157.96 | 0 |
| Hazard-rate | ~detectability class | 21162.81 | 4.852733 |
| Hazard-rate | ~hermite | 21185.44 | 27.47821 |
| Hazard-rate | ~cosine | 21185.44 | 27.47821 |
| Hazard-rate | ~polynomial | 21185.44 | 27.47821 |
| Hazard-rate | ~1 | 21185.44 | 27.47821 |
| Half-normal | ~cosine | 21193.64 | 35.67672 |
| Half-normal | ~polynomial | 21237.64 | 79.68529 |
| Half-normal | ~detectability class + group size | 21240.26 | 82.29678 |
| Half-normal | ~hermite | 21242.98 | 85.01553 |
| Half-normal | ~detectability class | 21243.19 | 85.22783 |
| Half-normal | ~1 | 21266.43 | 108.4673 |
| Roe Deer | | | |
| Key function | Formula | AIC | ∆AIC |
| Hazard-rate | ~detectability class + group size | 9688.111 | 0 |
| Hazard-rate | ~detectability class | 9692.245 | 4.133737 |
| Hazard-rate | ~hermite | 9718.419 | 30.3086 |
| Hazard-rate | ~cosine | 9718.419 | 30.3086 |
| Hazard-rate | ~polynomial | 9718.419 | 30.3086 |
| Hazard-rate | ~1 | 9718.419 | 30.3086 |
| Half-normal | ~detectability class + group size | 9737.106 | 48.99535 |
| Half-normal | ~detectability class | 9737.294 | 49.18303 |
| Half-normal | ~cosine | 9738.816 | 50.70492 |
| Half-normal | ~hermite | 9744.298 | 56.18685 |
| Half-normal | ~polynomial | 9761.627 | 73.51579 |
| Half-normal | ~1 | 9761.627 | 73.51579 |
| Fallow Deer | | | |
| Key function | Formula | AIC | ∆AIC |
| Half-normal | ~detectability class | 3197.52 | 0 |
| Hazard-rate | ~hermite | 3197.575 | 0.054453 |
| Hazard-rate | ~cosine | 3197.575 | 0.054453 |
| Hazard-rate | ~polynomial | 3197.575 | 0.054453 |
| Hazard-rate | ~1 | 3197.575 | 0.054453 |
| Hazard-rate | ~detectability class | 3198.036 | 0.515737 |
| Half-normal | ~hermite | 3198.068 | 0.547442 |
| Half-normal | ~cosine | 3198.068 | 0.547442 |
| Half-normal | ~polynomial | 3198.068 | 0.547442 |
| Half-normal | ~1 | 3198.068 | 0.547442 |
| Half-normal | ~detectability class + group size | 3199.128 | 1.607455 |
| Hazard-rate | ~detectability class + group size | 3200.034 | 2.5136 |


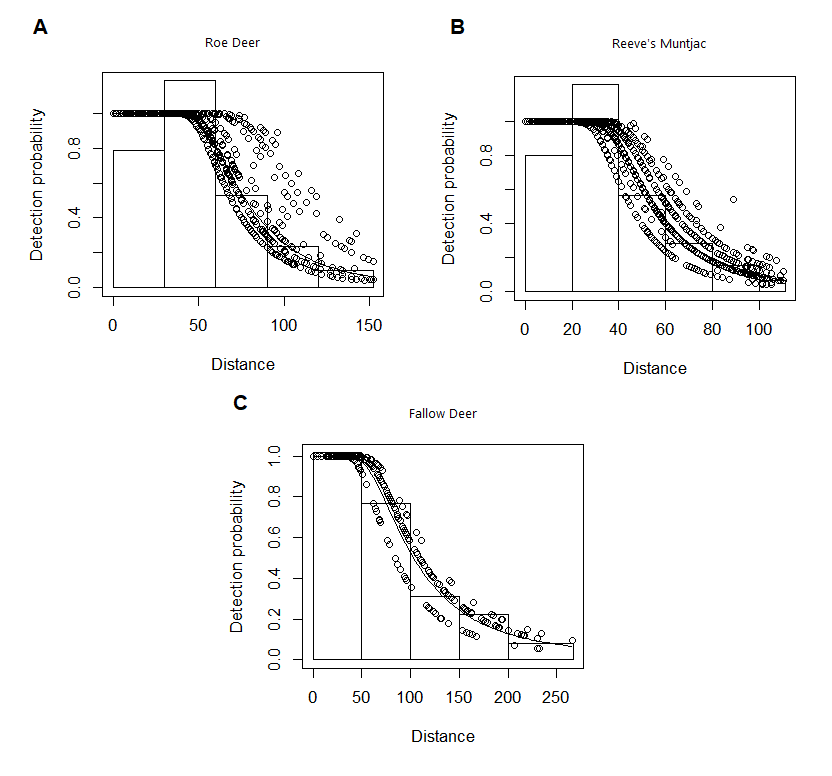


Figure S2: Species-specific detection functions. Selected species-specific detection functions of Reeve’s Muntjac (A), Roe Deer (B), and Fallow Deer (C) of distance sampling data pooled across 2011 to 2017. Continuous lines represent the average detection function, while hollow points represent observations along the categories of the covariate adjustments: for Reeve’s Muntjac, and Roe Deer these consider detectability class and group size covariates; for Fallow Deer, detectability class.

*Density surface models year-specific parameters.*— Separately for each deer species, the selected detection functions and adjustments were used to predict the density in each transect segment. Pooling the detection function across years would not allow fitting of species-specific annual DSMs, therefore, separate detection functions were fitted each year using a consistent detection function formulation based on the exploration of pooled data (see above). The number of observations differed annually (Table S3), as a result of changes in deer densities and also sampling effort (Table S1) but were sufficient to allow fitting of robust annual species-specific detection functions allowing annual species-specific Density Surface Models to be generated (Table S3).

Table S3. Annual species-specific number of observation and Effective Strip Width and average species-specific density. For each year number of group observations, Effective Strip Width (ESW), and average species-specific density of deer across the surveyed blocks for each species Reeve’s Muntjac, Roe Deer, and Fallow Deer are reported.

| Year | Reeve’s Muntjac | | | Roe Deer | | | Fallow Deer | | |
| --- | --- | --- | --- | --- | --- | --- | --- | --- | --- |
|  | N. obs | ESW | Deer /km^2^ | N. obs | ESW | Deer /km^2^ | N. obs | ESW | Deer /km^2^ |
| 2011 | 231 | 57 | 12.6 | 95 | 94 | 3.7 | 42 | 23 | 6.3 |
| 2012 | 205 | 54 | 11.1 | 93 | 86 | 3.6 | 30 | 142 | 0.6 |
| 2013 | 440 | 61 | 12.2 | 182 | 76 | 4.2 | 55 | 24 | 3.6 |
| 2014 | 295 | 54 | 9.3 | 149 | 83 | 3.3 | 47 | 114 | 0.8 |
| 2015 | 273 | 55 | 9.3 | 129 | 97 | 2.4 | 47 | 90 | 1.2 |
| 2016 | 397 | 60 | 11.4 | 163 | 77 | 3.9 | 53 | 120 | 0.9 |
| 2017 | 662 | 70 | 15.2 | 260 | 83 | 4.8 | 45 | 142 | 0.5 |

Table S4: Species-specific Coefficients of Variation (*CV*) of detection functions, GAM, and total *CV*.

|  | *CV* Detection function | *CV* from GAM | Total *CV* |
| --- | --- | --- | --- |
| Roe Deer 2011 | 0.12 | 0.32 | 0.34 |
| Roe Deer 2012 | 0.09 | 0.21 | 0.23 |
| Roe Deer 2013 | 0.06 | 0.1 | 0.11 |
| Roe Deer 2014 | 0.06 | 0.09 | 0.11 |
| Roe Deer 2015 | 0.05 | 0.12 | 0.14 |
| Roe Deer 2016 | 0.08 | 0.1 | 0.13 |
| Roe Deer 2017 | 0.04 | 0.07 | 0.08 |
| Reeve’s Muntjac 2011 | 0.05 | 0.32 | 0.33 |
| Reeve’s Muntjac 2012 | 0.06 | 0.15 | 0.16 |
| Reeve’s Muntjac 2013 | 0.04 | 0.06 | 0.07 |
| Reeve’s Muntjac 2014 | 0.04 | 0.07 | 0.08 |
| Reeve’s Muntjac 2015 | 0.05 | 0.07 | 0.09 |
| Reeve’s Muntjac 2016 | 0.04 | 0.06 | 0.08 |
| Reeve’s Muntjac 2017 | 0.03 | 0.04 | 0.05 |
| Fallow Deer 2011 | 0.66 | 0.34 | 0.74 |
| Fallow Deer 2012 | 0.11 | 1.62 | 1.62 |
| Fallow Deer 2013 | 0.64 | 0.27 | 0.69 |
| Fallow Deer 2014 | 0.13 | 0.19 | 0.23 |
| Fallow Deer 2015 | 0.26 | 0.2 | 0.33 |
| Fallow Deer 2016 | 0.08 | 0.19 | 0.21 |
| Fallow Deer 2017 | 0.33 | 0.19 | 0.38 |

Fallow Deer annual observations were lower compared to the observations collected for the other species and detection function less robust (Table S4). Fallow deer are herding animals (average group size across years =5.0, *SD* =3.4), with much wider home ranges (average across winter and spring home ranges = 219ha, Borkowski and Pudełko 2007) while Reeve’s muntjac and roe deer are relatively sedentary (average across winter and spring home ranges =19.4ha and 73.9ha, Chapman et al. 1993) and occur only singly or in small groups (average group size across years: Reeve’s Muntjac = 1.2, *SD* 0.4; Roe Deer = 1.7, *SD* 0.8). Consequently, when collecting distance sampling thermal imaging data for Fallow Deer detecting (or missing) a single herd introduces greater sampling error than for the two smaller species. Annual density surfaces were generated for Fallow Deer as a running mean across the focal and adjacent years (for 2011: mean of 2011-12; for all other years 2012-2017: the running average of three years centred on the focal year as data from 2018 were also available). For the two smaller species robust annual density surfaces were generated, owing partly to their greater overall frequency.

**Literature Cited**

Borkowski J., Pudełko M. 2007. Forest habitat use and home-range size in radio-collared

fallow deer. Annales Zoologici Fennici. 44:107–14.

Buckland S.T., Anderson D.R., Thomas L., Burnham K.P., Laake J.L., Borchers D.L. 2001. Line transects. In: Buckland S.T., editor. Introduction To Distance Sampling: Estimating Abundance of Biological Populations. Oxford: Oxford University Press; p. 104–137.

Chapman N.G., Claydon K., Claydon M., Forde P.G., Harris S. 1993. Sympatric populations

of muntjac (*Muntiacus reevesi*) and roe deer (*Capreolus capreolus*): a comparative analysis of

their ranging behaviour, social organization and activity. Journal of Zoology. 229:623–640.

Elenga G., Bonenfant C., Péron G. 2020. Distance sampling of

duikers in the rainforest: dealing with transect avoidance. PLoS One 15: e0240049.

Miller, D. L. 2016. Package ‘Distance.’

https://cran.rproject.org/web/packages/Distance/Distance.pdf

Miller D.L., Burt M.L., Rexstad E.A., Thomas L. 2013. Spatial models for distance sampling data: recent developments and future directions. Methods in Ecology and Evolution. 4:1001–1010.

R core Team. 2018. R: A Language and Environment for Statistical Computing, Vienna, Austria. [www.R-project.org/](http://www.r-project.org/).

Wäber K., Dolman P.M. 2015. Deer abundance estimation at landscape-scales in heterogeneous forests. Basic and Applied Ecology. 16:610–620.

Zini V., Wäber K., Dolman P.M. 2022. Relation of pine crop damage to species-specific density in a multi-ungulate assemblage. European Journal of Forest Research. 141: 489–502.
